# Single-Layer or Double-Layer Intestinal Anastomosis: A Systematic Review of Randomized Controlled Trials

**DOI:** 10.7759/cureus.46697

**Published:** 2023-10-08

**Authors:** Donatus K Okafor, Gitika Katyal, Gursharan Kaur, Hafsa Ashraf, Adi Prasad Bodapati, Ayesha Hanif, Safeera Khan

**Affiliations:** 1 Research, California Institute of Behavioral Neurosciences and Psychology, Fairfield, USA

**Keywords:** “post-operative complications”, anastomotic leak, bowel anastomosis, double-layer, single-layer, colorectal anastomosis, intestinal anastomosis

## Abstract

Several malignant and benign indications may necessitate bowel resection. Despite the emergence of newer techniques, the hand-sewn technique remains popular for the reestablishment of intestinal continuity after resection. This method can achieve anastomosis in one or two layers. Some studies have suggested that the single-layer technique has several potential benefits compared to its rivals while simultaneously maintaining a comparable efficacy and safety profile. Previous reviews have failed to recommend either of these methods over the other due to a lack of high-quality evidence. This review aims to establish which technique provides the best outcomes by reviewing recent relevant trials and comparing both methods. We conducted a systematic review of randomized controlled trials (RCTs) using the Preferred Reporting Items for Systematic Reviews and Meta-Analyses (PRISMA) checklist. A database search of PubMed, Google Scholar, Embase, and the Cochrane Central Register of Controlled Trials (CENTRAL) ultimately returned nine randomized trials published between 2003 and 2023 comparing single-layer intestinal anastomosis (SLIA) and double-layer intestinal anastomosis (DLIA) that fit the inclusion criteria. Overall, results show a dearth of robust trials, and the included studies displayed variable eligibility criteria and materials used for anastomosis. The available evidence, however, does suggest that neither technique is inferior in terms of preventing post-operative complications, but SLIA is less expensive and quicker to perform. The evidence is, however, limited, and further high-quality research is needed.

## Introduction and background

The establishment of principles for bowel anastomosis marked a remarkable feat for the surgeon, enabling the re-establishment of bowel continuity after resection. Since then, little has changed, and the time-tested hand-sewn technique remains a popular option even in the face of emerging new technologies such as stapling devices and anastomotic rings [1]. It is affordable and widely available, and the vast majority of surgeons share a common familiarity with the technique. Furthermore, as studies have failed to demonstrate the superiority of the newer emerging methods [1-3] and anastomotic leaks (AL) remain a dreaded complication [4], surgeons have often reverted to reliable hand-sewn anastomosis when faced with challenging high-risk surgery [5]. It is, however, whether to use the single-layer or double-layer approach for hand-sewn anastomosis that remains the source of debate.

The original two-layer approach, or double-layer intestinal anastomosis (DLIA), has historically been considered the conventional approach [1]. It typically consists of an inner layer of absorbable sutures, followed by a seromuscular layer of non-absorbable sutures. Some authors have suggested that long durations of surgery and higher costs of surgery have shifted emphasis towards the alternate option, single-layer intestinal anastomosis (SLIA) [3,5], which provides a potentially simpler, less expensive, and less time-consuming alternative that requires only a single layer of interrupted or continuous sutures [5].

The feared potential complication, regardless of technique, is the AL, which unfortunately complicates up to 5% of intestinal anastomosis. When it occurs, it increases morbidity and mortality and is associated with prolonged stays in hospitals and increased healthcare costs [4]. Theoretically, SLIA is associated with less tissue handling [6], and there is a lower likelihood of tissue strangulation as suture tension is more evenly distributed around the gut wall, thereby producing less ischemia and perhaps fewer anastomotic leaks [7,8]. However, previous reviews and meta-analyses have not been able to confidently establish such a difference.

A 2012 Cochrane review concluded that although SLIA showed promise of faster surgeries with a similar complication profile as DLIA, the quality of evidence comparing both techniques was suboptimal [6]. Following the authors' recommendation that further, more robust research was required, multiple newer trials have attempted to compare both approaches [9]. However, no reviews have accounted for these trials, leaving this important clinical question mostly unanswered. Our aim was to perform a systematic review of recent randomized controlled trials (RCTs), comparing the safety and efficacy of both techniques with a view to developing recommendations to guide practice and further research.

## Review

Methods

We performed a systematic review of randomized control trials (RCTs) using the guidelines from the Preferred Reporting Items for Systematic Reviews and Meta-Analyses checklist (PRISMA) [10]. We have chosen to include only RCTs as this represents the best quality evidence to guide treatment. Without proper randomization, researchers may preferentially allocate patients to a particular treatment arm of a trial depending on presentation, with the implication that more high-risk patients may undergo a particular procedure. Similar issues arise from retrospective reviews [11].​​​

Search Sources and Strategies

An electronic search of the following databases was carried out: PubMed, Google Scholar, Embase, and the Cochrane Central Register of Controlled Trials (CENTRAL). The search was limited to studies published between 2003 and 2023. The search strategy included the following keywords: ‘anastomosis’, ‘colorectal’, ‘rectal’, ‘single-layer’, ‘anastomotic’ leak, ‘intestinal’ ‘ileal’, ‘ileocolic’ ‘dehiscence’, ‘stricture’, ‘one-layer’, double-layer’, ‘two-layer’, ‘two layers’, ‘single-layer anastomosis’, ‘esophageal anastomosis’, ‘gastric anastomosis’, ‘small bowel anastomosis', ‘gastrointestinal anastomosis', and ‘double-layer anastomosis’. We also searched reference lists of identified articles for other potentially relevant studies. On PubMed, we combined the Booleans ‘AND’, 'OR', and ‘NOT’ with Medical Subject Headings (MeSH) to isolate more relevant articles. We filtered our search to include only publications dated between 2003 and 2023. All databases were last consulted on July 26, 2023.

Screening

The identified articles were collected and transferred to the reference management tool, Mendeley. The screening was carried out by two researchers working independently. Duplicates were first identified and removed. Then we screened the titles and abstracts, followed by full-text screening. Disagreements were resolved in consultation with a third researcher.

Eligibility Criteria

Only RCTs published between 2003 and 2023 and available in English were included. In order to meet the inclusion criteria, studies must compare the single-layer with the double-layered hand-sewn anastomotic techniques to establish gastrointestinal continuity. Studies comparing stapling devices with hand-sewn techniques, non-RCT studies such as retrospective studies, and those that involved anastomosis of extra-intestinal gastrointestinal structures such as pancreatic duct surgeries and esophageal studies were excluded. Grey literature, non-human studies, and ex-vivo studies were all removed.

Data Extraction

Two authors independently retrieved selected data using an Excel spreadsheet that was tested on the first two studies, then edited and standardized to extract data from the remaining trials. Conflicts were reconciled by a consensus among all the investigators. Data extracted included the study title, dates of the trials, population, sample size, inclusion and exclusion criteria used, and surgical techniques and sutures used. The occurrence of postoperative AL will be considered the primary outcome, as it is the most feared complication. Secondary outcomes include length of hospital stay, presence of stenosis or intestinal obstruction, cost of anastomosis, time taken to perform anastomosis (duration of anastomosis), and mortality.

Risk of Bias Assessment

All included studies were RCTs and were assessed using the revised Cochrane risk-bias tool for randomized trials (ROB2) by two reviewers working separately. Risk is assessed across five domains, namely, randomization, deviations from intended outcomes, missing outcome data, measurement of the outcome, and selection of the reported results [11]. Harmonization of the reviewers’ assessment was done, and where there were differences in the assessment of bias in any domain, arbitration was provided by a third researcher.

Where some information necessary to fully assess the risk of bias was missing, this was classified as 'some concerns'. Trials with concerns in more than two domains were ranked as having an overall high risk of bias if the concerns significantly reduced confidence in the results.

Results

**Figure 1:**
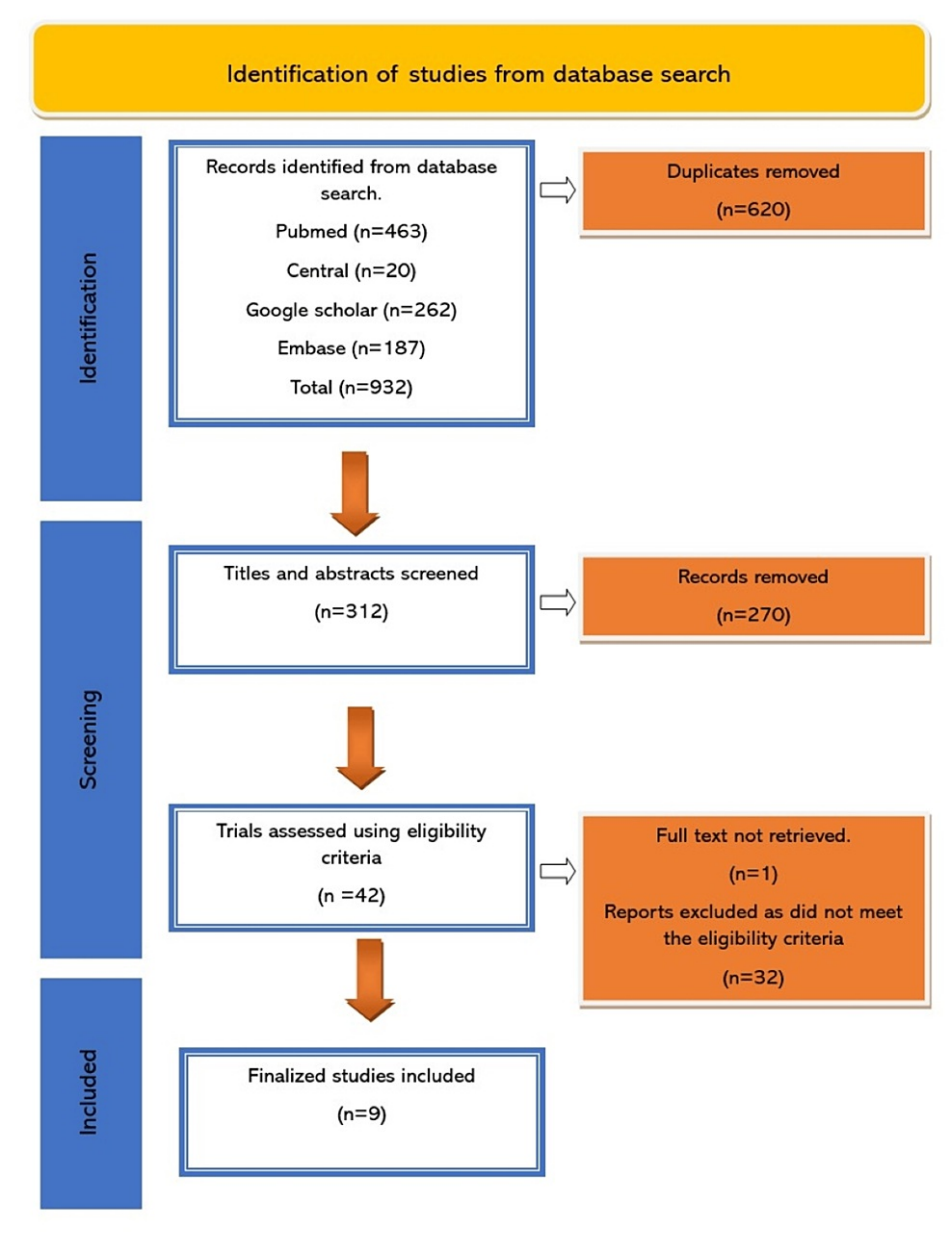
PRISMA flow diagram for literature search and selection PRISMA: Preferred Reporting Items for Systematic Reviews and Meta-Analysis, n: number of studies

A total of 932 papers were identified by the initial search. Using Mendeley, 620 duplicates were removed. Screening of titles and abstracts excluded a further 270 articles. One potential article had to be removed as full texts were unavailable for review. This meant that 42 studies underwent full-text assessment, at which point we excluded a further 32 articles, leaving a finalized number of nine articles. The PRISMA flow diagram showing the process of scrutinizing the identified articles is presented in Figure 1.

Characteristics of Included Trials

All researchers unanimously agreed that the nine trials included met the set-out inclusion criteria. The earliest trial started recruitment in 2004, while the earliest publication was a different trial in 2013. The sample size range was 50 to 250 patients.

Criteria for inclusion and exclusion, age, and outcomes assessed varied between trials. Only two studies included children; one study included only children aged four months to 14 years [12], whereas in another trial, the lower age limit was set at 12 years [13]. Some trials excluded patients with anemia [3,14], or those requiring anastomosis to peculiar sites such as gastric and duodenal [3,5,12,13], as well as rectal anastomosis [3,5,13], or middle and distal rectal anastomosis [15]. Table 1 summarizes the characteristics of the included studies.

**Table 1:** Characteristics of included studies AL: anastomotic leak, ASA: American Society of Anesthesiologists, DLIA: double-layer intestinal anastomosis, DM: diabetes mellitus, Hb: hemoglobin, HTN: hypertension, ITU: intensive therapy unit, SLIA: single-layer intestinal anastomosis, SNOSE: sequentially numbered, opaque, sealed envelopes, n: number, NG: not given, TPN: total parenteral nutrition

Study	Country	Year	Size (n)	Follow-up period	Characteristics	Inclusion criteria	Exclusion criteria	Outcome measures	Comments
Kar et al. [3]	India	2013-2015	97	14 days	Random number generator with permuted block sizes of 10.	Age 18-65 years; hemodynamically stable with Hb > 8 g/dl	Gastric, duodenal, or rectal anastomosis; comorbidities, cachexia, or intraoperative events that may influence recovery	Anastomotic leak; intraabdominal abscess; pelvic collection; abdominal distension; hospital stay; cost of suture materials; duration of anastomosis; post-operative return of bowel function	Patients who had SLIA had a significantly shorter duration for establishment of anastomosis and post-operative hospital stay compared to those with DLIA. Other parameters were comparable. The randomization method was good, the study is limited by small sample size.
Aniruthan et al. [5]	India	2016	60	3 months	Permuted block randomization; SNOSE concealment; open label.	Elective and emergency procedures; end-end or side to side anastomosis; age 18 yrs and above	<18 yrs of age; anastomosis including the rectum, or duodenum; anastomosis to the stomach	Anastomotic leak: length of hospital stay; mortality; surgical site infections	No significant differences were found in any of the outcomes measured in this study.
Ahmad et al. [12]	Pakistan	2016	60	Till discharge	Randomization -Lottery	Only benign indications for resection; age of 1month - 14 years	Prior intestinal anastomosis; gastric or duodenal anastomosis; malnutrition; malignant disease	Length of hospital stay	This is the only study that focused specifically on anastomosis in the paediatric population. Unfortunately, it only tested for length of hospital stay and rates of AL were not analyzed here. DLIA group was found to have spent significantly longer hospital days than SLIA group. The authors, however, did not specify if the surgical team in charge of discharging the patient were blinded or aware of the interventions the patients had received. This could lead to a bias in potentially discharging patients from one treatment arm quicker.
Garude et al. [13]	India	2007-2019	145	3 months	Alternating allotment	All hand-sewn anastomosis in patients 12 yrs or older	Patients younger than 12 years of age; anastomosis involving the rectum or duodenum	Surgical cost; Hospital stay; anastomotic leak; abdominal abscess.	According to this study, SLIA had better outcomes for mean duration of anastomosis and cost of surgery while maintaining comparable efficacy in preventing anastomotic leaks as DLIA. Despite being one of the studies with the largest sample size, we judged the potential for bias to be high as an alternative allotment method was used for patient allocation.
Gondil et al. [14]	India	2020-2022	50	NG	Random number generator	Age 18-65 yrs; hemodynamically stable	Terminally ill patients; pregnant patients.	Duration of procedure; anastomotic leak; hospital stay.	There was significantly less time of anastomosis associated with SLIA than DLIA. No difference was noticed in other parameters of interest. Although there was good randomization, sample size is the limiting factor here.
Herrle et al. [15]	Germany	2004-2012	252	3 months	Central computer random sequence generation	Age at least 18 years, Elective surgeries, only Ileo-colic or Colo-colic anastomosis were included.	ASA score > 3; missing or no informed consent; middle or lower third rectal anastomosis; surgeon deciding that one of the two interventions was inappropriate.	Anastomotic leak; duration of anastomosis; frequency of anastomotic strictures; mortality.	This represents the largest study included in this review. It was a multi-center study conducted approximately 8 yrs and found comparable outcomes for SLIA and DLIA in all outcomes of interest except for duration of anastomosis where SLIA was actually deemed to have performed better.
Moeen et al. [16]	Pakistan	2012	200	7 days	No randomization technique was given beyond stating that the participants were “randomly allocated.”	Benign diseases	Malignant diseases	Anastomotic leak	This study was the only one to report a significant difference in occurrence of anastomotic leaks between the two interventions. The researchers found SLIA to be more efficacious than DLIA in this regard. However, this study is limited in several aspects. Although it contains one of the largest sample sizes in this review, it contained no information on how allocation or concealment was done. There were also concerns about the lack of information as to the criteria used to make the diagnosis of leaks in the study and due to lack of published pre-trial protocol, concerns about the reported outcomes.
Shah et al. [17]	India	2021	60	NG	Patients were alternatively allotted to one intervention or the other.	Age 18-65 years; Emergency or elective; Hemodynamically stable, Hb >8 g/dl; jejuno-jejunal, jejunoileal and ileo-ileal anastomosis; ileocolic and Colo-colic anastomosis; and stoma closure	Gastric, duodenal, and rectal anastomosis; immunocompromised patients; consent not given by the patient.	Anastomotic leaks; duration of anastomosis; mortality.	Mostly comparable outcomes between the two interventions. However, shorter duration of anastomosis was achieved with the one-layer technique. Poor randomization and small sample size are the main limiting qualities here.
Singh et al. [18]	India	2021	60	NG	Chit and box method for randomization into the two groups.	Hemodynamically stable	Comorbidities - HTN, DM, immunodeficiency; bleeding disorders; stapled procedures; sepsis; patients on TPN; patients in the ITU; other complications from surgery or anesthesia.	Anastomotic leak; duration of anastomosis; abscess and fistula formation; wound infection; duration of hospital stay; mortality.	All parameters were comparable except duration of anastomosis where SLIA performed better. Issues with sample size, concealment, lack of specified criteria for leak definition limit the quality of this evidence.

Surgical Technique

Seven of the included trials provided detailed accounts of the surgical techniques used. The approach and materials for achieving either type of anastomosis varied slightly between the trials. In performing SLIA, researchers used either continuous sutures, interrupted sutures, or both. Other variations included the size of sutures and absorbable versus non-absorbable sutures, which are summarized in Table 2.

**Table 2:** Techniques and materials for single- and double-layer anastomosis used in the included studies DLIA: double-layer intestinal anastomosis, SLIA: single-layer intestinal anastomosis, PDS: polydioxanone suture

Study	Technique SLIA	Technique DLIA
Kar et al. [3]	Continuous 3/0 PDS sutures; all layers except mucosa	Inner layer - continuous 3/0 polyglycolic acid
		Outer layer - Lembert sutures interrupted 3/0 silk sutures
Aniruthan et al. [5]	Continuous or interrupted. 3/0 polyglycolic acid and polypropylene were used 4-6 mm from the wall of the edge, 5 mm apart incorporating all layers except the mucosa	Inner layer - continuous or interrupted using 3/0 polyglactin
		Seromuscular/Lembert - interrupted using 3/0 silk sutures
Garude et al. [13]	Continuous 3/0 polypropylene 4-6 mm from the wall of the edge, 5 mm apart. All layers sutured except the mucosa.	Inner layer - continuous 3/0 polyglactin
		Seromuscular layer - Lembert sutures interrupted 3/0 silk sutures with inversion of the inner edges
Herrle et al. [15]	Continuous 4/0 PDS sutures applied 4 to 5 mm from the edge with inversion of the mucosa	Inner layer - continuous 5/0 PDS sutures
		Seromuscular layer - continuous 4/0 or 5/0 PDS sutures
Shah et al. [17]	Interrupted 3/0 polyglactin stitches 5 mm apart	Inner layer - continuous 3/0 polyglactin
		Outer layer - interrupted 3/0 silk sutures
Singh et al. [18]	Interrupted 3/0 polyglycolic acid sutures excluding the mucosa	Inner layer - continuous 3/0 polyglycolic acid and other absorbable sutures
		Outer layer - interrupted runs of 3/0 silk suture

Appraisal of Studies

We used the revised Cochrane risk-of-bias tool for randomized trials (RoB2) to assess the risk of bias in each of the selected trials [11].

The trials showed varying degrees of limitations. One trial sought only to measure the length of hospital stay [12], but it is not clear whether the assessors were knowledgeable of the assigned intervention and if they oversaw discharging the patients, thereby raising concerns of potential bias in measuring outcomes. The alternating technique of allocation used in Garude et al. [13] and Shah et al. [17] undermines the randomization process as the patient's recruitment may be affected by the recruiter’s or participants’ knowledge of the sequence. Singh et al. used the Chit and Box method for randomization, but poor concealment may give the researchers knowledge of the sequence [18]. No information as to randomization was provided by Moeen et al. beyond stating that their patients were randomly allocated [16].

Given that neither Singh et al. nor Moeen et al. provided a definition for anastomotic leaks used in their studies [16, 18], we were concerned that different diagnostic criteria may have been applied to different patients as it is possible for researchers to define anastomotic leaks differently [19].

Furthermore, we did not find prospectively registered pre-trial protocols for some of the studies. Questions were therefore raised as regards the choice of outcomes reported or omitted, especially for those trials where only a few parameters were analyzed with the exclusion of others [12,16,17].

**Figure 2:**
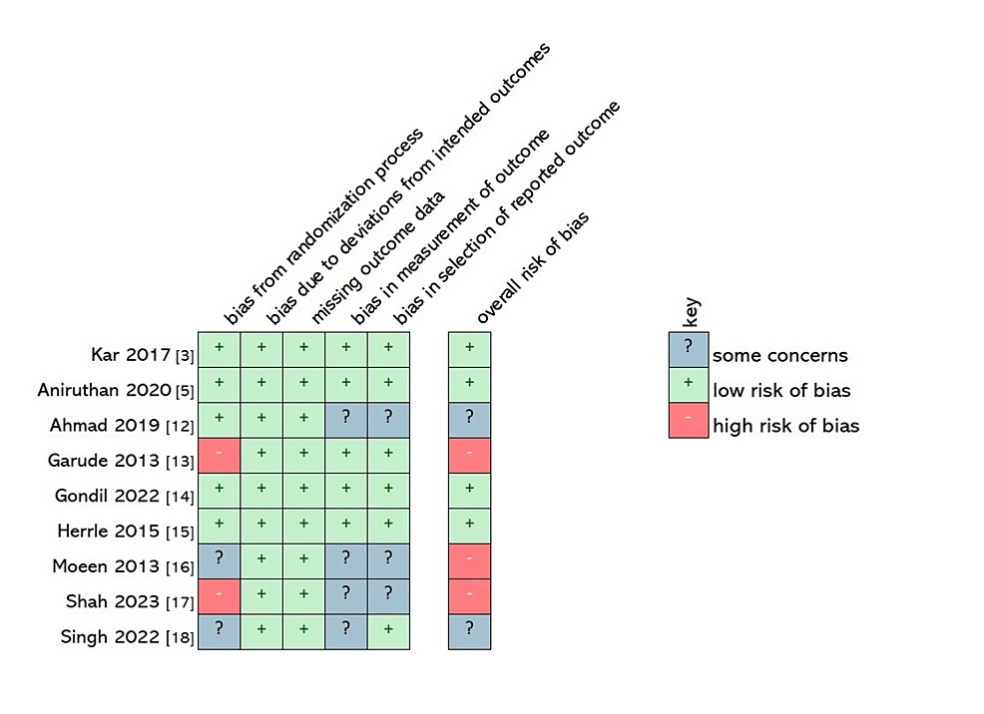
Revised risk of bias (ROB 2) appraisal of included trials Applying the revised risk of bias tool to appraise the included trials [3,5,12-18]

**Figure 3:**
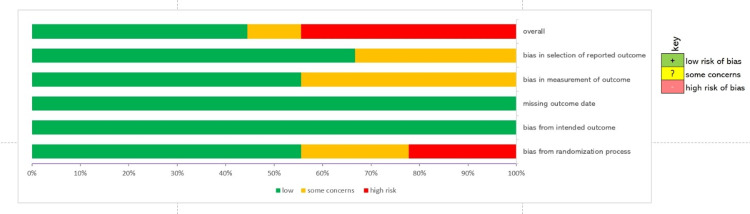
Proportion of studies classified into different risk of bias groups

The risk of bias in each domain of each trial is summarized in Figure 2. Figure 3 shows the proportion of studies classified as low-risk, with some concerns and a high risk of bias in each domain.

Anastomotic Leaks

**Figure 4:**
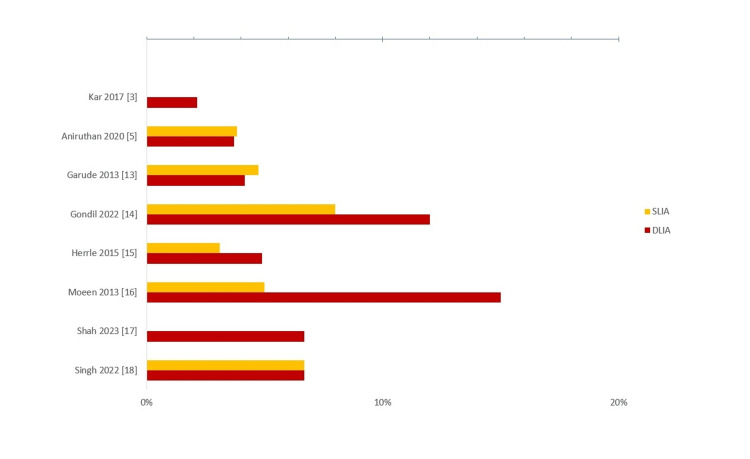
Proportion of participants developing post-operative anastomotic leaks in each trial Occurrence rates of anastomotic leak among participants in the included trials [3,5,13-18] DLIA: double-layer intestinal anastomosis, SLIA: single layer intestinal anastomosis

Eight out of nine included trials assessed for anastomotic leaks. The four best-quality studies reported no statistical differences between the techniques. Only the study by Mooen et al. showed significantly lower rates of anastomotic leaks in the SLIA intervention arm [16]. This study, however, was judged to have a high risk of bias. The proportion of patients with anastomotic leaks in each intervention arm is shown in Figure 4.

Duration of Anastomosis and Cost of Materials

Six different trials compared SLIA and DLIA in terms of the duration taken to achieve anastomosis. The mean duration ranged from 9.50 minutes to 19.7 minutes for SLIA and 19.50 to 30.00 minutes for DLIA. SLIA was significantly shorter in duration in all studies, including three with a low risk of bias [3,14,15]. Both studies that analyzed cost, one with low risk and the other with high risk of bias, determined that SLIA was significantly cheaper than DLIA [3,13].

Stenosis

No studies demonstrated a significant difference in rates of stenosis between the techniques.

The study by Herrle et al. showed two cases of stenosis among the SLIA group and no cases among the patients who had DLIA. This relationship was, however, not statistically significant [15].

Length of Hospital Stay and Mortality

The majority of good-quality studies showed no difference in length of hospital stay following either intervention, except for Kar et al., where patients who had SLIA were discharged at significantly faster rates than those who received DLIA (p<0.001) [3]. This finding was corroborated by Ahmad et al., who demonstrated similar findings in the pediatric population, albeit in a lower-rated trial [12].

Similarly, no studies showed any significant difference in rates of mortality.

Table 3 shows the quantitative results from each of the included studies. Table 4 summarizes the primary and secondary outcomes and the quality of the supporting evidence for each outcome.

**Table 3:** Summary of findings from included studies Summary of findings from included studies [3,5,12-18]. Continuous variables are presented as mean ± standard deviation (SD) or median (range)^c^ Rates of anastomotic leaks and mortality are presented as percentages (%) of the number of patients in each treatment arm per study. Cost of sutures is presented as a number (n) - which represents price. SL: single layer, DL: double layer. ^a^Indian rupees. ^b^Currency not indicated. ^c^Median values (range), rather than mean ± SD. ^†^Mean given but SD not calculated. *Significant findings. NG: not given by study.

	Mean age (yrs.)		Mean duration of anastomosis (minutes)		Total cost of sutures (n)		Mean hospital stay (days)		Anastomotic leak (%)		Mortality (%)	
Author	SL	DL	SL	DL	SL	DL	SL	DL	SL	DL	SL	DL
Kar et al. [3]	42.96 ± 3.76	42.63 ± 3.34	15.12 ± 2.27	24.38 ± 2.26*	564^a^	480^a^	5.90 ± 1.43*	7.29 ± 1.89*	0.0%	2.13%	2.00%	2.12%
Aniruthan et al. [5]	47.67 ± 14.35	50.67 ± 14.56	NG		NG		8.33 ± 3.72	8.89 ± 3.78	3.84%	3.70%	1.92%	3.70%
Ahmad et al. [12]	5.57 ± 4.00	5.40 ± 3.46	NG		NG		4.5† *	6.10†*	NG	NG	NG	NG
Garude et al. [13]	33.00^†^	33.00^†^	9.50^†^	19.50^†^*	298^b^	390^b*^	12	12	5.47%	4.17%	NG	NG
Gondil et al. [14]	NG		18.85 ± 1.55	27.90 ± 2.01*	NG		7.48 ± 1.88	8.01 ± 2.55	8.00%	12.00%	0.00%	4.00%
Herrle et al. [15]	67.7 ± 11.1	64.6 ± 13.4	18.00 (1–49)^c^	24.00 (8–50) ^c^*	NG		9 (5–128)^c^	8.5 (4–29)^c^	3.10%	4.90%	1.55%	0.00%
Moeen et al. [16]	33.40 ± 6.07	32.94 ± 5.28	NG		NG		NG	NG	5.00%	15.00%*	NG	NG
Shah et al. [17]	39.26^†^	45.40^†^	14.35 ± 2.55	21.43± 1.99	NG		NG	NG	0.00%	6.67%	3.33%	0.00%
Singh et al. [18]	38.60^†^	34.63^†^	19.57 ± 2.25	30.00 ±2.59*	NG		8.97 ± 3.08	8.93 ± 2.61	6.67%	6.67%	0.00%	3.33%

**Table 4:** Outcomes assessed by the included trials SLIA: single-layer intestinal anastomosis, DLIA: double-layer intestinal anastomosis, AL: anastomotic leak

Outcome assessed	Number of trials (n)	Number and quality of trials showing each finding
AL	8	Seven studies - Four good quality trials [3,5,14,15], one moderate quality trial [18], and two low quality trials [13,16] suggested that rates of anastomotic leaks were comparable. One low quality trial [16] found SLIA to be the safer option. No trials suggested that DLIA was better than SLIA in preventing anastomotic leaks.
Time to achieve anastomosis	6	All six studies found SLIA to be the quicker option to perform including three good quality - [3,14,15], one moderate quality - [18], and two poor quality trials [13,17].
Cost comparison	2	Both studies: one good quality [3], and one low quality [13], found SLIA to be the cheaper option.
Length of hospital stay	7	Five studies including three good quality trial [5,4,15], one moderate quality [18], and one poor quality trial [13] showed comparable results between both interventions. Two trials, a good quality trial [15], and a moderate quality trial [18] found significantly shorter duration of hospital stay among SLIA patients. DLIA was the better option in none of the included studies.
Stenosis	2	Both studies found comparable values. One was a good quality trial [15].
Mortality	6	All studies found mortality rates to be comparable. This included four high quality trials [5,14,15], one moderate quality [18] and one low-quality study [17]. No studies found significantly lower rates of deaths with either intervention.

Discussion

The potential benefits of SLIA continue to drive research to establish its safety and efficacy in comparison to DLIA. This review summarizes the last 20 years of randomized trials on this topic, including nine studies and a total of 984 patients. In our review, most studies took place in India (six) and Pakistan (two), in contrast to previous reviews that were dominated by European trials [6]. Stapled anastomosis offers an alternative that may reduce both the interest and the recruiting power of studies comparing handsewn techniques as the popularity of staplers increases. Paradoxically, retrospective and prospective studies have shown that surgeons will preferentially perform hand-sewn techniques on patients with a perceived higher burden of disease, especially in acute care settings. Interestingly, despite this surgeon-derived bias, failure rates are equivalent [20] or doubled when stapling is done [21]. It therefore remains relevant to establish a universal first-choice technique for hand-sewn anastomosis. The interest in this topic among global researchers is crucial, as findings from more developed countries may not always be universally applicable [22].

Many of the studies in this review excluded anastomosis involving the stomach [3,5,12,13,15,17], the duodenum [3,5,12,13,17], and/or the entire rectum [5,13,17], or part of the rectum [15]. This was done to increase homogeneity as the stomach is more vascular and less likely to bleed [5], the duodenum and the rectum may be difficult to fully mobilize [5], and the rectum has a higher risk of leaks. It is also becoming increasingly popular to use stapled anastomosis for the rectum [15]. Unsurprisingly, trials that did not exclude the rectum had the highest overall rates of anastomotic leaks [14,16,18]. Excluding rectal anastomosis may have also limited the recruiting power of the trials. Future trials should include high-risk anastomosis and analyze the best technique to use in such cases. No good-quality studies have been done on children, and most trials have excluded the elderly population. Age, systemic conditions, and immunocompromise have been thought to affect the occurrence of anastomotic leaks. Similarly, modifiable factors such as smoking, alcohol use, and obesity are known to affect healing. These trials did not test for the impact of these factors on gastrointestinal healing.

We also noticed a relative variability in techniques among the studies. In performing single-layer anastomosis, some centers described an interrupted technique, while others chose a continuous technique for anastomosis, and yet others used both. Continuous technique may take less time and fewer sutures, but again, high-quality evidence is lacking. However, it is not obvious that using continuous and/or interrupted sutures introduces significant heterogeneity, as both techniques have demonstrated clinical equipoise [23]. The preferred use of continuous sutures for single-layer anastomosis among surgeons appears to be from a technical and time-saving point of view rather than proven superior efficacy.

Eight studies were included that assessed anastomotic leaks. As in previous systematic reviews [1,6], we found that SLIA and DLIA had comparable rates of anastomotic leaks in most trials. Only the article by Moeen et al. reported a significant difference between the techniques, with SLIA having a lower incidence of leaks [16]. Although this was one of the larger trials with 200 participants, it ultimately showed an overall high risk of bias. The best available evidence therefore suggests that rates of anastomotic leaks are similar.

Previous reviews have shown that SLIA tests are better than DLIA in terms of the duration of anastomosis and the cost of surgery. We included six studies, including three good-quality trials, that assessed the time taken to establish anastomosis. In all the studies, the single layer took significantly less time in our review [3,14,15]. The surgeon must delineate layers of the intestine before applying sutures to each layer in the double-layer technique, and this technique is thought to require higher technical skill [6]. Similarly, two studies analyzed the cost of suture materials, and in both cases, DLIA was the significantly more expensive option [3,13]. The vast majority of studies used a different suture material for each of the layers in their double-layer anastomosis [3,5,13,17,18], which means that surgeons inevitably must expend an extra suture when performing the DLIA. Herrle et al. used PDS sutures for both the seromuscular and inner layers of their double-layer anastomosis, but this study did not compare the costs of surgery [15].

It is documented that surgical trials are notoriously difficult to conduct [9], and it may be difficult to answer these questions in any single surgical trial. Issues with sample size, blinding, and measurement outcome have been known to plague the quality of surgical trials. Recruiting patients who have presented in emergency situations may not always be practical; there may be issues with patient preference or uncommon conditions; and apparently, only a third of surgery trial patients can be adequately blinded [24,25]. The population size in our review ranged from 50 to 252 patients, which is only slightly higher than what had been tested in the Cochrane review in 2012 [6]. The research by Herrle et al., which was the largest multicenter trial included, calculated a sample size of 768 but eventually had to settle for 252 due to slow uptake over eight years across four centers. This multi-center trial also reported difficulties keeping surgeons motivated to recruit patients in all the centers involved [15]. Poor randomization reduced the quality of various studies, especially where the alternating allocation technique was used, which means that the sequence may be predictable. Interestingly, some of the larger studies were compromised by issues of randomization and a lack of pre-trial protocols and definitions [16]. Given the challenges with blinding and sample recruitment, future researchers should emphasize good randomization, concealment, harmonization of interventions, and published pre-trial protocols to improve the quality of the evidence.

It would likely require a large multi-center study, meticulously designed and standardized, to adequately test this hypothesis [9,15]. Such a trial should crossmatch for systemic conditions and lifestyle choices that may affect the development of anastomotic leaks, the location of anastomosis along the intestine, suture materials, and continuous versus interrupted suture techniques. Until such a trial is concluded, however, the evidence has remained consistently limited but has consistently suggested that SLIA and DLIA have comparable outcomes.

## Conclusions

The current data, albeit problematic, suggests that performing the more expensive and potentially time-consuming DLIA offers no significant advantages over SLIA. The level of evidence on this topic has always been poor, and research over the last few years has done little to change that. At least until better evidence is available, SLIA may be considered first-line for intestinal anastomosis given better technical, time, and economic considerations. Further research should develop a recorded pre-trial protocol that is consistently followed, address issues with randomization, and aim to compare both techniques in low-risk and high-risk scenarios.
